# Isolation of *Bordetella avium* and Novel *Bordetella* Strain from Patients with Respiratory Disease

**DOI:** 10.3201/eid1501.071677

**Published:** 2009-01

**Authors:** Amanda T. Harrington, Jaime A. Castellanos, Tomasz M. Ziedalski, Jill E. Clarridge, Brad T. Cookson

**Affiliations:** University of Washington, Seattle, Washington, USA (A.T. Harrington, J.E. Clarridge III, B.T. Cookson); VA Puget Sound Health Care System, Seattle (J.E. Clarridge III); Baylor College of Medicine, Houston, Texas, USA (J.A. Castellanos); Western Washington Medical Group, Everett, Washington, USA (T.M. Ziedalski)

**Keywords:** Bordetella avium, Bordetella, pneumonia, dispatch

## Abstract

*Bordetella avium* is thought to be strictly an avian pathogen. However, 16S rRNA gene sequencing identified 2 isolates from 2 humans with respiratory disease as *B. avium* and a novel *B. avium–*like strain. Thus, *B. avium* and *B.* *avium*–like organisms are rare opportunistic human pathogens.

Several *Bordetella* species have been associated with respiratory disease in humans. Although *B. avium* is thought to be strictly an animal pathogen that causes tracheobronchitis in wild and domesticated birds (*1*,*2*), infections in birds share many of the clinical and histopathologic features of disease in mammals caused by *B. pertussis* and *B. bronchiseptica* (*3*). Human cases of respiratory disease associated with *B. avium* have only recently been reported in patients with cystic fibrosis (*4*). We describe 2 isolates, *B. avium* and a novel strain resembling *B. avium*, isolated from 2 patients with pneumonia, thereby demonstrating that *B. avium* and *B. avium–like* organisms are opportunistic human pathogens.

## The Cases

Patient 1, a 68-year-old man with left lower lobe pneumonia and hemoptysis, sought care at a community hospital. The patient had a history of end-stage renal disease requiring hemodialysis, chronic obstructive pulmonary disease (COPD), long-term tobacco use, and ischemic cardiomyopathy being treated with anticoagulation. He had a 10-day history of increasing shortness of breath and cough associated with mild hemoptysis. The initial chest radiograph demonstrated an extensive left-sided infiltrate, which progressively worsened; the patient ultimately required endotracheal intubation and mechanical ventilation. Bronchoscopy showed purulent secretions in the left mainstem bronchus, complete obstruction of the bronchus, and frothy secretions in the right airways. Gram-stained bronchoalveolar lavage (BAL) fluid showed many polymorphonuclear leukocytes but no organisms. Routine bacterial culture of the fluid isolated a pure culture that was phenotypically identified by API NFT rapid test strip (bioMérieux, Hazelwood, MO, USA) as *B*. *avium.* Antimicrobial drug–susceptibility testing performed using Etest (AB Biodisk, Solna, Sweden) resulted in the following MICs: ceftriaxone 2 μg/mL, azithromycin 4 μg/mL, piperacillin/tazobactam 0.125/4 μg/mL. The patient was initially treated with azithromycin and ceftriaxone and completed empiric therapy with piperacillin/tazobactam and vancomycin. By day 7, the patient’s respiratory status improved enough that he could be extubated. Follow-up chest radiographs showed substantial resolution of the left-sided infiltrates, and on day 11, the patient was discharged to a rehabilitation care facility.

Patient 2, a 61-year-old homeless man, was admitted to the Houston DeBakey Veteran Affairs Medical Center with a 4-month history of productive cough and a 1-month history of sporadic hemoptysis. He denied fever, chills, shortness of breath, and chest pain. His medical history included COPD, pulmonary tuberculosis, and long-term use of tobacco and alcohol. HIV ELISA result was negative. Chest radiographs and computed tomography scan showed marked emphysematous changes with bullae in the posterior left lobe, numerous calcified granulomas compatible with old granulomatous disease in the left lung, and pleural thickening and reaction in the left apex. A lobulated 2-cm soft tissue density in 1 of these cavities was also identified. No acute infiltrates were seen. The patient began treatment for COPD and for suspected pulmonary tuberculosis (ethambutol 1,600 mg/day, rifampicin 600 mg/day, pirazinamide 1,500 mg/day, and isoniazid 300 mg/day). Gram-stained BAL fluid showed many polymorphonuclear leukocytes and small gram-negative rods. Routine bacterial culture of BAL fluid isolated a pure culture that was identified as *Bordetella* spp. but that could not be identified to the species level by API NFT rapid test strip. Susceptibility testing was performed by using the Kirby-Bauer disk-diffusion method. Although no susceptibility guidelines for *Bordetella* spp. have been established, the isolate appeared to be sensitive to amikacin, trimethoprim-sulfamethoxazole, gentamicin, ceftazidime, and imipenem and resistant to ampicillin, ampicillin/sulbactam, and aztreonam. An oral cephalosporin drug was added to the patient’s regimen. Three sputum samples were negative for acid-fast bacilli, and treatment for tuberculosis was discontinued. The patient was discharged 9 days after hospital admission.

Isolates of nonlactose fermenting, small, gram-negative rods were recovered from BAL fluid by using trypticase soy agar supplemented with 5% sheep blood, chocolate agar, and MacConkey agar incubated at 35°C in 5% CO_2_. The isolate from patient 1 was phenotypically consistent with *B. avium* (catalase positive, oxidase positive, nitrate reduction negative, urease negative). The isolate from patient 2 was phenotypically inconsistent with all other well-characterized *Bordetella* spp. (catalase positive, oxidase positive, nitrate reduction positive, urease negative) and could not be phenotypically identified to the species level.

Sequence analysis was performed by PCR targeting the 16S rRNA gene as previously described (*5*). Samples were prepared by using the ABI PRISM BigDye Terminator Cycle Sequencing Kit (Applied Biosystems, Foster City, CA, USA), and sequencing reactions were performed by using the ABI PRISM 3100 Analyzer according to manufacturer’s instructions. Sequences were aligned by using Sequencher software and were submitted to GenBank BLAST database. The sequence from patient 1 was genetically identical to *B. avium* type strain ATCC 35086 (477 bp evaluated, 100% sequence similarity), and the sequence from patient 2 was related to *B. avium* type strain ATCC 35086 (509 bp evaluated, 98% sequence similarity [submitted as GenBank accession no. EU352642]). Several 16S rRNA gene sequences of type strains from the genus *Bordetella* were retrieved from GenBank, and a phylogenetic tree was constructed by using a neighbor-joining algorithm with *Achromobacter xylosoxidans xylosoxidans* as the outgroup (Figure). Concise alignment of the sequence from patient 2 showed 11-bp and 15-bp differences compared with *B. avium* (type strain ATCC 35086) and *B. trematum* (type strain DSM 11334), respectively. Thus, it represents a novel uncharacterized strain most closely related to *B. avium* (i.e., *B. avium–*like; Figure). Both the genotype of *B. avium* and the novel strain are distinguishable from other *Bordetella* spp. according to 16S rRNA gene sequencing of the first 500 bp (≈2% bp difference). This finding is in contrast to the indistinguishable sequences of *B. pertussis*, *B. parapertussis*, *B. bronchiseptica*, and the closely related *B. homlseii,* which can be a limitation of 16S rRNA gene sequencing for the genus *Bordetella*, particularly when only the first 500 bp are evaluated (Figure).

**Figure:**
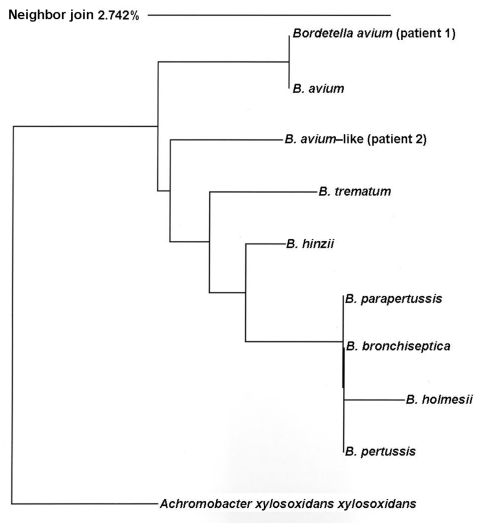
16S rRNA dendrogram. Phylogenetic tree of relationships among *Bordetella* spp. inferred on the basis of aligned 16S rRNA gene sequences from type strains (first 500 bp); a neighbor-joining algorithm with *Achromobacter xylosoxidans xylosoxidans* is used as an outgroup. *B. avium,* ATCC35086; *B. bronchiseptica,* ATCC19395; *B. hinzii,* ATCC51783; *B. holmseii,* ATCC51541; *B. parapertussis,* ATCC15311; *B. pertussis,* ATCC9340; *B. trematum,* DSM11334. Scale bar indicates percentage genetic distance.

## Conclusions

Although the first report of human disease associated with *B. avium* was recently described in patients with cystic fibrosis (*4*), the role of *B. avium* in respiratory infection as opposed to colonization in patients with cystic fibrosis is unclear. The cases in this report provide a more direct clinical association of human respiratory infection and *B. avium* and *B. avium*–like organisms. Each of the 2 isolates was obtained in pure culture from BAL fluid. Although neither patient was conventionally immunocompromised (e.g., no HIV, hematologic disorders, or immunosuppressive therapy), each was an older person who had underlying pulmonary problems along with other medical conditions, and each belonged to a patient population typically susceptible to opportunistic infections. Each patient had signs and symptoms clinically consistent with respiratory syndromes caused by *Bordetella,* and each responded to treatment. Clinicians should be aware that human infections with *B. avium* may pose some antimicrobial drug treatment challenges. A previous report demonstrated that cultures of *B. avium* were consistently resistant to penicillin and cefuroxime but susceptible to mezlocillin, piperacillin, gentamicin, amikacin, and cefoperazone (*6*). Although *B. avium* has been shown to share several virulence factors with *B. pertussis*, *B. parapertussis*, and *B. bronchiseptica*, it does not carry genes that encode pertussis toxin or adenylate cyclase toxin (*7*). *B. avium* and *B. avium*–like organisms have yet-unidentified virulence factors, which may contribute to their ability to cross over from an animal host to an opportunistic human pathogen.
